# The effect of dietary phytochemicals on nuclear factor erythroid 2-related factor 2 (Nrf2) activation: a systematic review of human intervention trials

**DOI:** 10.1007/s11033-020-06041-x

**Published:** 2021-01-30

**Authors:** Tom Clifford, Jarred P. Acton, Stuart P. Cocksedge, Kelly A. Bowden Davies, Stephen J. Bailey

**Affiliations:** 1 Department of Sport and Exercise Sciences, Manchester Metropolitan University, Manchester, M15 6BH UK; 2 School of Sport, Exercise and Health Sciences, Loughborough University, Loughborough, LE11 3TU UK

**Keywords:** Polyphenols, Antioxidants, Oxidative stress, Redox balance, Nutrition

## Abstract

We conducted a systematic review of human trials examining the effects of dietary phytochemicals on Nrf2 activation. In accordance with the PRISMA guidelines, Medline, Embase and CAB abstracts were searched for articles from inception until March 2020. Studies in adult humans that measured Nrf2 activation (gene or protein expression changes) following ingestion of a phytochemical, either alone or in combination were included. The study was pre-registered on the Prospero database (Registration Number: CRD42020176121). Twenty-nine full-texts were retrieved and reviewed for analysis; of these, eighteen were included in the systematic review. Most of the included participants were healthy, obese or type 2 diabetics. Study quality was assessed using the Cochrane Collaboration Risk of Bias Assessment tool. Twelve different compounds were examined in the included studies: curcumin, resveratrol and sulforaphane were the most common (*n* = 3 each). Approximately half of the studies reported increases in Nrf2 activation (*n* = 10); however, many were of poor quality and had an unclear or high risk of bias. There is currently limited evidence that phytochemicals activate Nrf2 in humans. Well controlled human intervention trials are needed to corroborate the findings from in vitro and animal studies.

**Supplementary information:**

The online version of this article (10.1007/s11033-020-06041-x) contains supplementary material, which is available to authorized users.

## Introduction

Phytochemicals, defined as plant metabolites, are ubiquitous in the human diet [1]. Indeed, several thousand different phytochemicals have been identified in commonly consumed plants [2]. These phytochemicals can be sub-divided into four higher order classes, based on their chemical structure: phenols and polyphenols, terpenoids, alkaloids, and sulphur containing compounds [2]. Although not deemed essential for health, numerous longitudinal studies report positive associations between the intake of phytochemicals and health, including a lowered risk of cardiovascular, neurodegenerative, and metabolic disease [3–8]. For example, in a large study of more than 5000 Finnish adults, higher intakes of flavonoids, the major class of polyphenols in the human diet, was associated with a reduced risk of heart disease [8]. Similarly, intake of anthocyanins, polyphenols present in many fruits, was associated with a reduced risk of myocardial infarction in ≥ 90,000 middle-older age women [7]. These findings have sparked significant interest in elucidating the wider health promoting potential of phytochemicals and resolving the molecular bases of such effects.

Several putative mechanisms for the potential health promoting effects of phytochemicals have been postulated, with their function as antioxidants receiving the most attention. Although many different definitions of antioxidants exist, they are recognized as agents that donate electrons to, and thereby stabilize, oxidants to prevent them from oxidizing other molecules [9, 10]. While some degree of oxidation is an important and necessary biological process, an excessive increase in reactive species (also referred to as free radicals) that exceeds antioxidant capacity leads to oxidative stress and the associated oxidative damage to proteins, lipids, DNA and other molecules [10, 11]. Hence, excess production of reactive species is implicated in the initiation and progression of several diseases [12, 13]. To protect against oxidative damage, cells are endowed with an antioxidant defense system, which includes various antioxidant enzymes including superoxide dismutase, catalase, and glutathione peroxidase [14]. Under normal conditions, endogenous antioxidants maintain cellular redox state by effectively scavenging radicals [9, 10]. However, exposure to a stressor that augments oxidant production (e.g., chronic disease, exercise, pollutants, injury) can overwhelm endogenous cellular antioxidant defenses [14]. In such circumstances, exogenous phytochemicals or dietary antioxidants like vitamin C and E (collectively referred to as non-enzymatic antioxidants) might be needed to maintain cellular redox status and offset protein, lipid, and DNA oxidation [9, 11].

The antioxidant effects of phytochemicals have been largely ascribed to radical scavenging [15, 16]. Indeed, phytochemicals, including curcumin, resveratrol, and quercetin have been shown to scavenge reactive species in vitro [17, 18]. In human studies, direct measurement of reactive species is more challenging due to their high reactivity and short biological half-lives [19]. Instead, antioxidant effects are often inferred by measuring tissue concentrations of oxidation products, such as protein carbonyls or F2-isoprostanes [19, 20]. Although findings are inconsistent [21], there is evidence that phytochemicals can lower systemic or cellular levels of lipid or protein oxidation in humans, suggestive of antioxidant effects [22–25].

Although the radical scavenging observed in vitro is often extrapolated and used to explain the antioxidant effects of phytochemicals in vivo, this contention has been questioned [26]. Perhaps the best evidence to undermine direct antioxidant effects of phytochemicals in vivo is the fact that most phytochemicals are extensively metabolized and have poor bioavailability, such that systemic concentrations typically reached after ingestion (~ 1 µmol/L) are significantly lower than the concentrations required to directly scavenge reactive species [26–28]. Instead, the major mechanism for the antioxidant or biological effects of phytochemicals in vivo is increasingly being attributed to their induction of the redox sensitive transcription factor, nuclear erythroid 2-related factor 2 (Nrf2) [26, 29].

In response to homeostatic challenges Nrf2 upregulates ~ 250 cytoprotective genes, many of which code for proteins with antioxidant, anti-inflammatory or phase 2 detoxifying functions [30]. Nrf2 is primarily regulated by cysteine-rich kelch-like ECH associated protein 1 (Keap-1), which sequesters Nrf2 in the cytosol through continual ubiquination [31, 32]. By reacting with cysteine residues on Keap-1, reactive species or electrophiles remove its repressive functions and enable Nrf2 to accumulate [33, 34]. After dissociating from Keap-1, Nrf2 translocates to the nucleus where it forms a heterodimer with musculoaponeurotic fibrosarcoma proteins to activate the antioxidant response element (ARE)-DNA sequence (also referred to as the electrophile response element) [32, 35].

The various mechanisms by which phytochemicals activate Nrf2 are still being unraveled; however, a few of these have been well-described in the literature. Indeed, two prominent mechanisms are that phytochemicals initiate the Nrf2-ARE pathway by modifying cysteine residues on Keap-1, either by acting as electrophilic Michael acceptors, many after biotransformation to reactive quinones, or by upregulating protein kinases that phosphorylate Nrf2 and facilitate its dissociation from Keap-1 [36–38]. These actions suggest, somewhat paradoxically, that phytochemicals may confer antioxidant effects indirectly and as a result of their initial pro-oxidant effects [26, 39]. Since the reactions needed to stimulate Nrf2 are possible at the low, sub-toxic concentrations typically reported for phytochemicals [26], this substantiates the notion that the antioxidant effects phytochemicals are more likely to be indirect. Specifically, activating Nrf2 will upregulate synthesis of several antioxidant enzymes [40–42]. Given that oxidative stress and low grade inflammation underlie the pathogenesis of many degenerative chronic diseases, Nrf2 has emerged as an attractive therapeutic target for health promotion and disease prevention [30, 43].

The Nrf2 inducing effects of various phytochemicals have been well-described in the literature with activation of the Nrf2-ARE axis now recognized as the major mechanism by which phytochemicals mitigate pro-oxidative and pro-inflammatory insults [27, 44]. However, most of the research reporting Nrf2 induction with phytochemicals has been conducted in pre-clinical cell culture or animal models. These studies typically use supra-physiological doses that are not achievable with normal dietary intakes. In the few studies that have assessed the effect of dietary phytochemicals on Nrf2 activation in humans, conflicting findings have been reported [45–47]. As such, there is currently no consensus on the effectiveness of phytochemicals to activate Nrf2 in humans. Thus, the aim of the present systematic review was to evaluate the extant literature and determine the impact of dietary phytochemicals on Nrf2 activation in humans. This systematic review was conducted according to the Preferred Reporting Items for Systematic Reviews and Meta-Analyses (PRISMA) guidelines [48]. Given the emerging beneficial health effects associated with Nrf2 activation, determining the effectiveness of dietary components on its activation is a critical step in understanding how the Nrf2-ARE pathway can be therapeutically harnessed.

## Methods

The protocol for this systematic review was pre-registered on the PROSPERO database (Registration Number: CRD42020176121) and reported according to the PRISMA guidelines [48].

### Search strategy

Medline, Embase and CAB abstracts were searched for articles from inception until March 2020. Our search strategy was based on a Population, Intervention, Comparator, Outcome, Study design (PICOS) methodology (available in the Online Supplementary Material). Using Boolean logic and truncations, a comprehensive list of terms and keywords were searched that linked “phytochemical” and “Nrf2”. Our terms and key words were adapted from similarly designed review articles [49, 50]. Because over 60 terms were entered, these are not reproduced here but are available in the Online Supplementary Material. Search terms were applied to the full texts; non-English studies were included in our search.

**Fig. 1:**
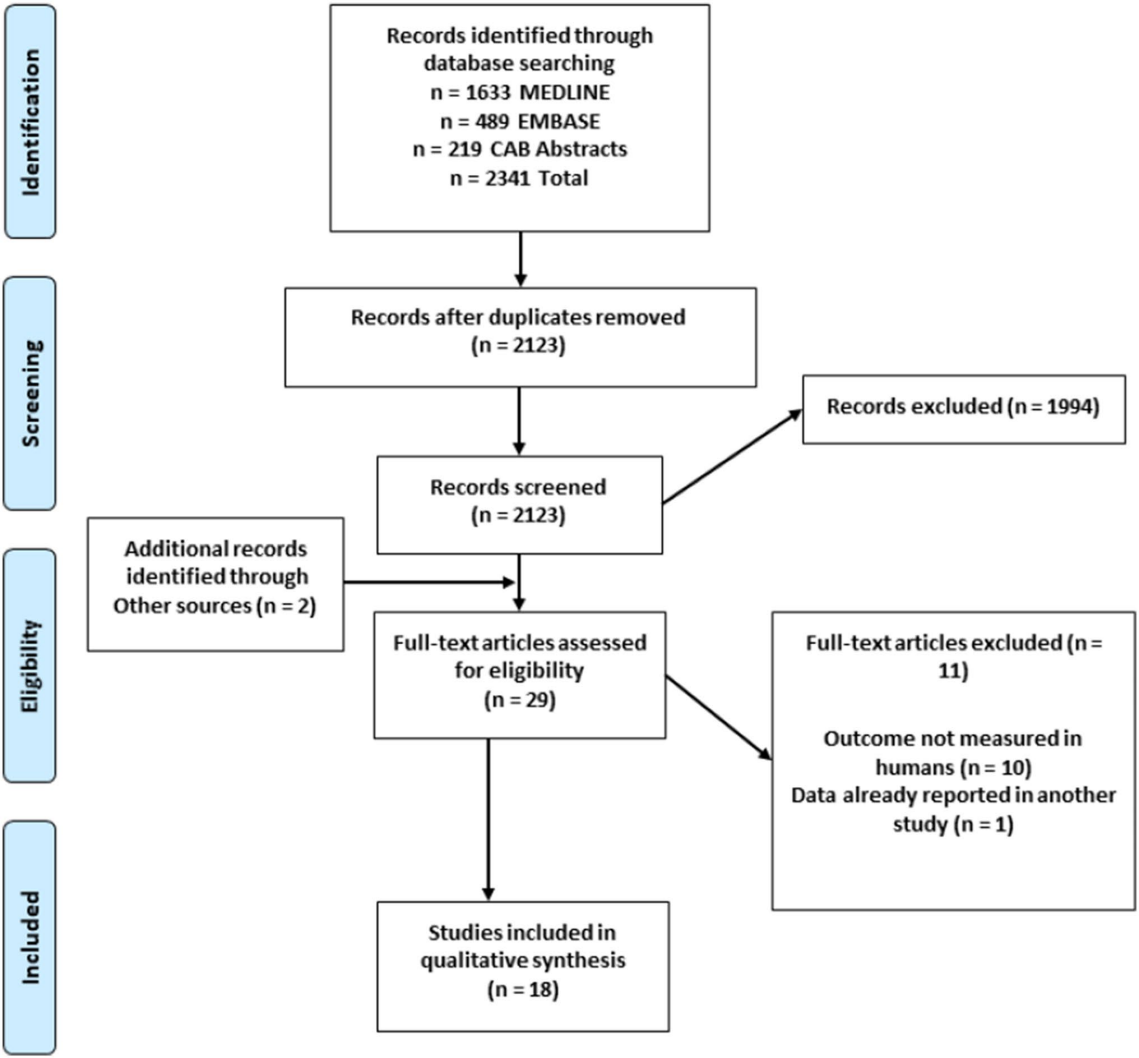
Flow diagram of the process used in selection of the trials included in this systematic review

The titles and abstracts of the articles were screened independently by two investigators (TC and KBD). The relevant full texts were retrieved to assess eligibility according to the criteria outlined below. All full-text articles included were searched manually for any additional studies; one was identified from this search [51]. Another [52], was identified from a Google Scholar search performed at this stage. A flow diagram of our search strategy is depicted in Fig. 1.

### Study selection

Inclusion criteria were: (1) adult participants (≥ 18 years); (2) administration of a dietary phytochemical (or a combination of phytochemical compounds); (3) reporting of pre to post changes in Nrf2 via any methods and in any tissue. No restrictions were added for study design, but we excluded studies in animals. The full text of articles deemed to meet these criteria were retrieved and independently screened for their eligibility by all investigators (see Online Supplementary Material for list of studies excluded). All investigators agreed on the articles to be included in the systematic review.

### Data extraction

Data were extracted by three authors (TC, SC and SB). The data were based on our PICOS (see Online Supplementary Material) and included type of participants and their age, the study design, the interventions, duration of intake, type of measurement (e.g., protein, gene expression etc.), tissue type, and outcome. Because of the wide inter-study heterogeneities in study design and interventions, a meta-analysis was deemed inappropriate. Extracted data is displayed in Table 1.

**Table 1:** An overview of studies included in the systematic review

Study	Subjects	Age (years)	Design	Intervention/comparator	Duration	Tissue	Measure	Change	Risk of bias^a^
Ghanim et al. [45]	Healthy males (*n* = 4) & females (*n* = 6)	37 ± 4	Crossover, placebo controlled	Resveratrol (100 mg) + grape (poly)phenols (75 mg/placebo (no details)	Single dose	PBMC	Total Nrf2 DNA-binding activity via EMSA	↔ 1 h ↑ 3 & 5 h post	Mod
Boettler et al. [61]	Healthy males (*n* = 27)	26 ± 1	Crossover	Coffee (500 ml/day; chlorogenic acid, 819.2 mg/L, N-methylpyridinium: 73.7 mg/L) / Low (poly)phenol diet	4 weeks	BLY	Total Nrf2 GE	↑ post	High
Magbanua et al. [63]	Male prostate cancer patients (*n* = 84)	60 ± 7	Three arms, randomized controlled trial, parallel	Lycopene (30 mg/day), fish oil (3 g/day)/placebo (no details)	12 weeks	Prostate tissue	Total Nrf2 GE	↑ at 12 weeks	Low
Volz et al. [51]	Healthy males (*n* = 29)	Range (20–44)	Crossover	Coffee (750 ml/day; Chlorogenic acid, 580.1 mg/L, N-methylpyridinium: 71.4 mg/L) / Low (poly)phenol diet	4 weeks	BLY	Total Nrf2 GE	↑ post	High
Kropat et al. [66]	Female Ileostomy probands (*n* = 5) and female healthy controls (*n* = 5)	N/R	Pre-post	Bilberry pomace (10 g/day)/N/A	Single dose	PBMC	Total Nrf2 GE	↔ 1 h post ↓ 2, 4 & 8 h	High
Perez-Herrera et al. [65]	Obese adults (*n* = 20)	56 (SD N/R)	Randomized controlled trial, crossover	0.45 ml/day of virgin olive oil (400 µg/ml phenols), mixed Seed oils (400 µg/ml phenols)/mixed seed oil)/0.45 ml of sunflower oil	Single dose	PBMC	Total Nrf2 GE & CTY & NC PL	↔ GE 4 h post ↓ NC PL 4 h post ↔ CTY PL 4 h post	Mod
Yubero-Serrano et al. [64]	Healthy females (*n* = 10) and males (*n* = 10)	67 ± 1	Three arms, randomized controlled trial, parallel	Med diet + co-enzyme Q10 (200 mg/day)/Med diet, western diet	4 weeks	PBMC	Total Nrf2 GE & CTY & NC PL	↔ GE 2 h ↓ GE 4 h ↑ CTY PL 4 h ↓ NC PL 4 h	Mod
Yang et al. [54]	Type 2 diabetic males (*n* = 8) & females (*n* = 6)	66 ± 3	Single arm, pre-post design	Curcumin (500 mg/day)/N/A *Con-med*	15–30^a^ days	PBL	Total Nrf2 PL	↑ post	High
Wise et al. [46]	COPD males (*n* = 54) & females (*n* = 35)	58 (median), SD N/R	Double-blind, randomized controlled trial, parallel	Sulforaphane (25 or 150 μmoles/day)/cellulose *Con-med*	4 weeks	AV-M, BRC-E, PBMC	Total Nrf2 GE	↔ post	Low
Saldanha et al. [58]	Non-dialyzed CKD males (*n* = 11) and females (n = 9)	62 ± 8	Double blind, randomized controlled trial, crossover	Resveratrol (500 mg/day)/wheat flour (500 mg/ day)	4 weeks	PBMC	Total Nrf2 GE	↔ post	Low
Jimenez-Osorio et al. [56]	Diabetic and non-diabetic CKD males (*n* = 56) and females (*n* = 40)	Nondiabetic: 40 ± 3	Double blind, randomized controlled trial, parallel	Curcumin (320 mg/day)/starch (320 mg/day)	8 weeks	PBMC	NRF2/ARE binding activity in NC	↔ post	Low
		Diabetic: 55 ± 2							
Duran et al. [60]	Healthy adults (*n* = 15)	Range (18–50)	Randomized controlled trial, crossover	broccoli sprout homogenate (200 g/day)/alfalfa sprout homogenate (200 g/day)	3 days	PBMC NEC	Total Nrf2 GE	↔ 4 h post	High
Kerns et al. [59]	Healthy adults (*n* = 4)	40 ± 4	Single blind, randomized controlled Crossover	Broccoli sprout extract (500 nmol/day of sulforaphane) in jojoba oil/jojoba oil only	1 week	Skin	Total & PY Nrf2 IMF	↑ post	High
Li and Zhang [62]	Ischemic stroke males (*n* = 125) & females (*n* = 75)	63 ± 10	Randomized controlled, parallel	Iso-flavones (80 mg/day)/ placebo (80 mg/day) *Con-med*	24 weeks	Blood	Total Nrf2 GE & PL	↑ GE & PL	Mod
Seyyedebra-himi et al. [57]	Type 2 diabetic males (n = 19) & females (*n* = 22)	57 ± 6	Double blind, randomized controlled, parallel	Resveratrol (800 mg/day)/cellulose (800 mg/day)	8 weeks	PBMC	Total Nrf2 GE	↑ post	Low
Jackman et al. [52]	Healthy males (*n* = 16)	67 ± 4	Double blind, randomized controlled, parallel	Cherry juice (60 ml/day; 540 mg anthocyanins)/placebo (60 ml/day)	2 weeks	SM	Total Nrf2 GE	↔ post	Mod
Bardagjy et al. [47]	Obese males (n = 4) & females (n = 16)	49 ± 15	Double blind, randomized controlled, crossover	Grape polyphenols (60 g/day; 297 mg of polyphenols) / placebo (60 g/day)	4 weeks	PBMC	Total Nrf2 GE	↔ 1 & 5 h post ↑ 3 h post	Low
Cheng et al. [55]	Healthy males (*n* = 7) & females (n = 5)	Range (18–27)	Single arm, pre-post design	Curcumin (4 g)/N/A	Single dose	PBLK	Total Nrf2 GE	↑ 4–6 h post	High

**Fig. 2:**
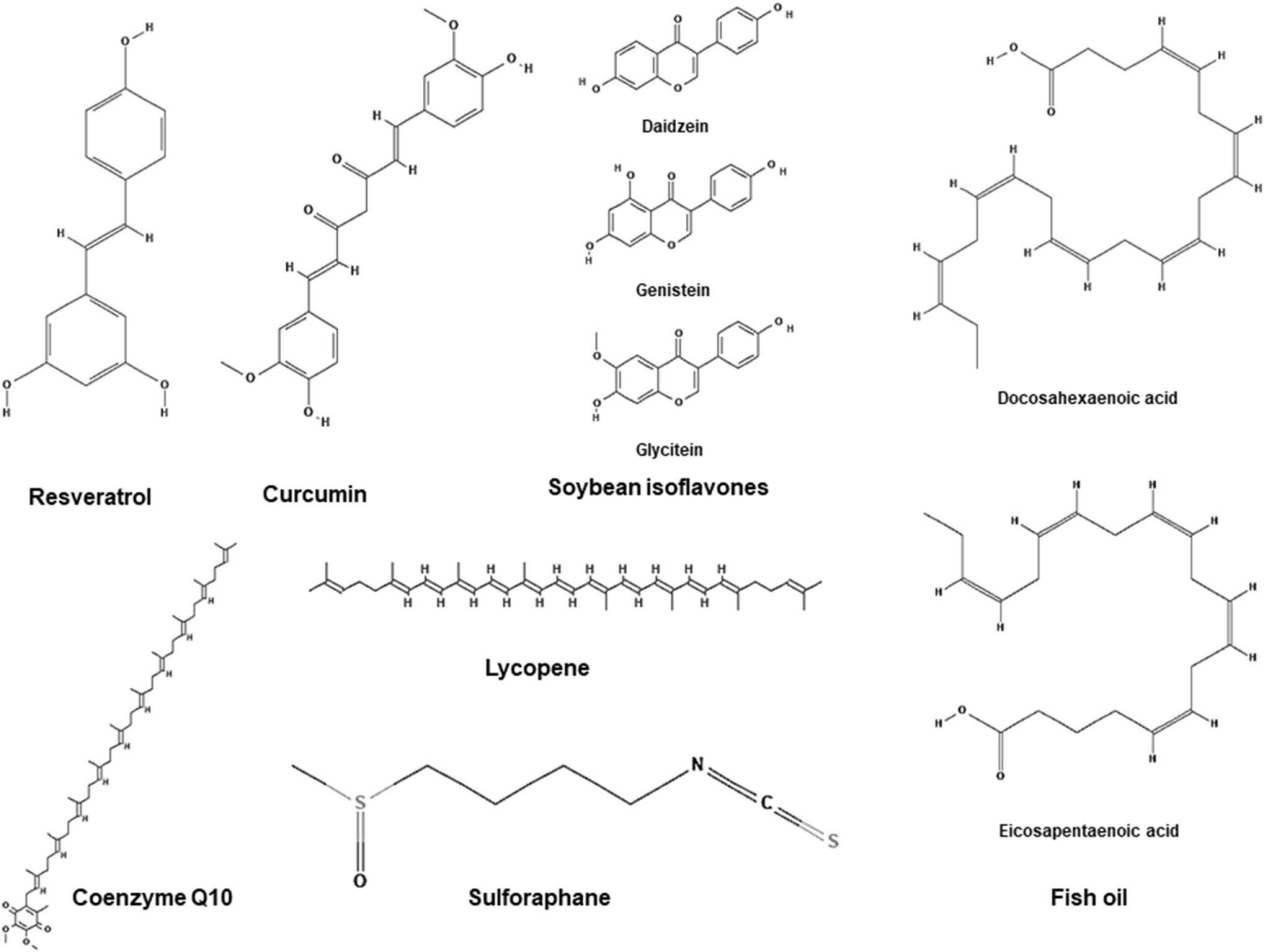
Chemical structures of main phytochemicals in the included studies. Structures from PubChem; references can be found in the Online Supplementary Material

**Fig. 3:**
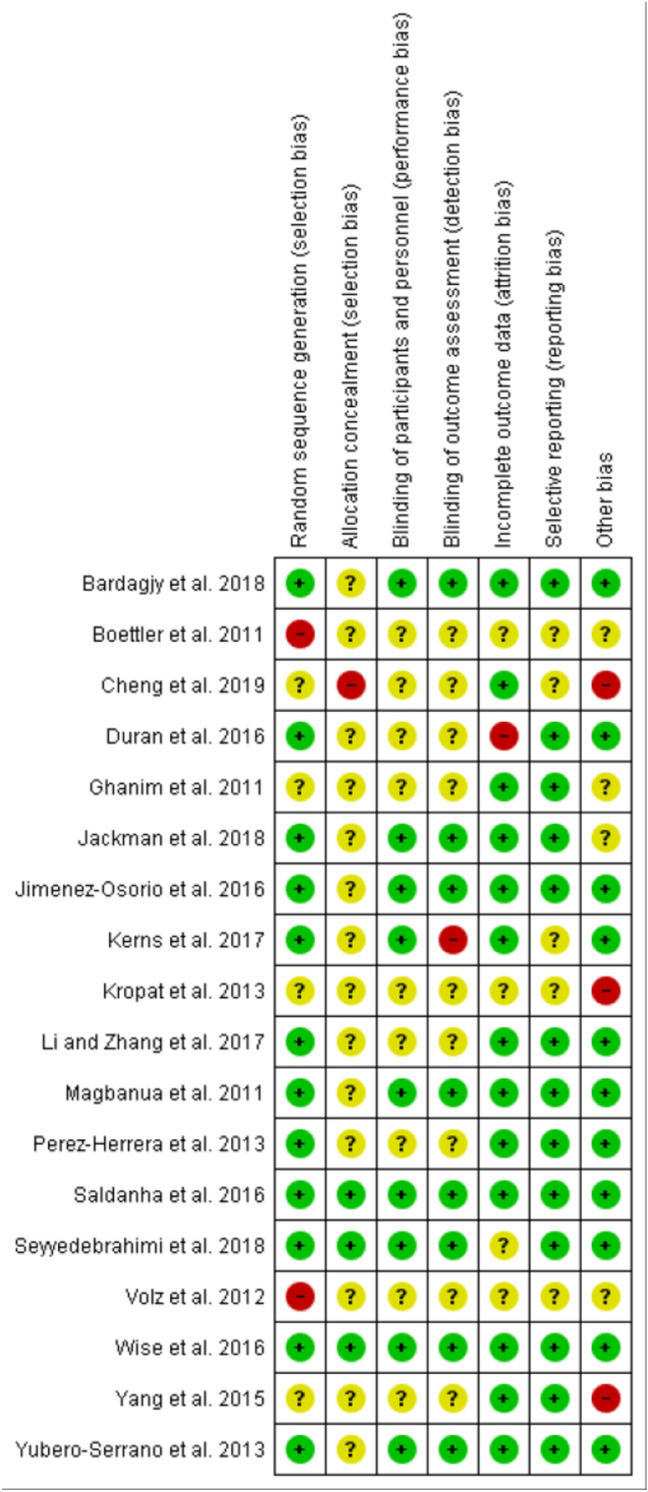
Risk of bias summary for incldued studies

This is a general score for illustrative purposes based on Figs. 2 and 3; Low = low risk of bias; Mod = moderate risk of bias; High = high risk of bias. Arrows indicate decrease, increase and no change

*ARE* antioxidant response element; *AV-M* alveolar macrophages; *BRC-E* bronchial epithelial cells; *PBLK* peripheral blood leukocytes; *BLY* blood lymphocytes; BSE; *CKD* chronic kidney disease; *Con-med* concomitant medications; *COPD* chronic obstructive pulmonary disease; *CYT* cytoplasmic fraction; *DB* double blind; *DNA-BA* DNA binding activity; *EMSA* electrophoretic mobility shift assay; *GE* gene expression; *IMF* immunofluorescence; *Med diet* Mediterranean diet; *NEC* nasal epithelial cells; *NC* nuclear fraction; *PBMC* peripheral blood mononuclear cells; *PBL* peripheral blood lymphocytes; *PL* protein levels; *PY* phosphorylated; *Nrf2* nuclear factor erythroid 2-related factor 2; *SD* standard deviation; *SM* skeletal muscle; *N/A* not applicable; *N/R* not reported

^a^Those with overt diabetic kidney disease consumed for 30 days; those without for 15 days

### Risk of bias

Study quality was assessed with the Cochrane Risk of Bias Tool [53] from Review Manager 5.3 (Cochrane Collaboration, UK). This was performed independently by two authors (TC and JA) and disagreements were resolved through discussion. Each study was rated as either low, unclear or high risk of bias according to the following criteria: random sequence generation, allocation concealment, blinding of participants and personnel, blinding of outcome assessment, incomplete outcome data, selective reporting, and “other” potential biases (e.g., conflicts of interest, inadequate study design).

## Results

### Search results

Results from our search strategy are presented in Fig. 1. We identified 2341 articles from three databases, which was reduced to 2123 after removing duplicates. After initial screening of abstracts and titles, we retrieved twenty-nine full texts; eleven were excluded and eighteen were deemed eligible and included in the review.

### Study characteristics

Table 1 summarizes the studies examining the effects of phytochemicals on Nrf2. Across the 18 included studies, 12 different phytochemicals were examined for Nrf2 activation. Only three individual phytochemicals were measured in more than one study; these were curcumin [54–56], resveratrol [45, 57, 58] and sulforaphane [46, 59, 60]. Other phytochemicals examined were soybean-isoflavones [62], lycopene [63], fish oil [63], and co-enzyme Q-10 [64]. The chemical structures of these phytochemicals are shown in Fig. 2. Some studies examined the effects of a whole food or fluid rich in phytochemicals; these included tart cherry juice [52], seed oils [65], bilberry [66], whole grape powder [47] and phytochemical enriched coffee [51, 61].

Three of the included studies were pretest–posttest quasi experimental designs with no placebo comparator [54, 55, 66]; seven studies employed a randomized crossover design [45, 51, 58–60, 65, 67]; eight employed a randomized parallel groups design [46, 52, 56, 57, 62–64].

In the included studies there was a total of 727 participants; average age ranged from 18 to 67 years. Nine trials were performed in healthy, disease free adults [45, 51, 52, 55, 59–61, 64, 66, 68]; three were in type 2 diabetics [54, 56, 57]; one was in chronic obstructive pulmonary disease patients [46]; two were in obese adults [65, 67]; one was in prostate cancer patients [63]; one was in ischemic stroke patients [62] and one was in chronic kidney disease patients [58]. Of these trials, only two were performed in adults ≥ 65 years of age [52, 64]. Of the seven trials in patients, only three recorded the intake of concomitant medications [46, 54, 62].

The length of the dietary intervention ranged from a single dose (*n* = 4) to 24 weeks; however, only one was longer than 12 weeks [62]. The most common duration was four weeks (*n* = 6). There was wide heterogeneity in doses used, within and between supplements (Table 1).

Most studies (*n* = 14) measured Nrf2 activation in peripheral blood cells [45, 51, 54, 55, 57, 58, 60, 62, 64–67, 69]. One measured Nrf2 in skeletal muscle [52]; one in prostate tissue [63]; one in skin [59]; one in nasal epithelial cells (as well as peripheral blood mononuclear cells; PBMCs) [60] and one in alveolar macrophages, bronchial epithelial cells and PBMCs [46].

Fifteen studies evaluated Nrf2 activation as gene expression [46, 47, 51, 52, 54, 55, 57–64, 66]; of these, three also measured protein levels [62, 64, 65]. One study measured protein levels only [54] and two studies measured Nrf2-DNA binding activity [45, 56].

### Phytochemicals examined in more than one trial

#### Coffee derived phytochemicals

Boettler et al. [61] reported that a four-week intake of 500 ml/day of coffee enriched with chlorogenic acid or n-methylpyridinium increased Nrf2 gene expression in 27 healthy young adults compared to a low polyphenol control diet. Similarly, Volz et al. [51] reported a significant 1.4 fold increase in Nrf2 gene expression in healthy individuals who consumed 750 ml of a phytochemical rich coffee per day for four weeks versus a low polyphenol control diet.

#### Curcumin

Yang et al. [54] showed that 500 mg of curcumin for 15 days significantly increased Nrf2 protein expression compared to baseline in type 2 diabetics. Similarly, in a pretest–posttest design in 12 healthy volunteers, Cheng et al. [55] found that 4 g of curcumin stimulated a ~ 1.3 fold increase in Nrf2 gene expression 4–6 h post-intake. By contrast, another study [56] found no change to Nrf2-ARE binding activity in kidney disease patients who consumed 320 mg/day of curcumin for eight weeks.

#### Resveratrol

A cocktail of resveratrol (100 mg) and grape polyphenols (75 mg) significantly increased Nrf2-ARE binding activity at 3 h (~ 150%) and 5 h (~ 100%) post consumption in 10 healthy adults [45] In a randomized, crossover study, Saldanha et al. [58] found no change in Nrf2 gene expression (− 0.27 fold) with a four week intake of resveratrol (500 mg/day) in kidney disease patients. Another study reported a significant increase in Nrf2 gene expression in diabetic patients consuming 800 mg/day of resveratrol for 2 months [57]. However, the reported values indicate that Nrf2 decreased with resveratrol supplementation (pre-intervention; 6.32 ± 1.05 vs. post-intervention 5.62 ± 1.35) (see “Discussion” below section).

#### Sulforaphane

Duran et al. [60] found no significant effect of a sulforaphane-rich broccoli sprout homogenate (200 g/day for three days) on Nrf2 gene expression (0.9 to − 12.6% change) in fifteen healthy young adults. Similarly, Wise et al. [46] found that four weeks of sulforaphane (25–150 μmoles/day) did not significantly modify Nrf2 gene expression (max average fold change 1.17) in chronic obstructive pulmonary disease patients. In contrast, Kerns et al. [59] reported an increase in total and phosphorylated Nrf2 expression from four healthy subjects who applied broccoli sprout extract (500 nmol/ml of sulforaphane) to their arm for seven days.

### Phytochemicals measured in single trials

Kropat et al. [66] found that an anthocyanin-rich bilberry extract (10 g) significantly decreased Nrf2 gene expression 2, 4 and 8 h post consumption (− 40 to − 60% of baseline) in female ileostomy probands and female healthy controls. Perez-Herrera et al. [65] examined whether adding 400 µg/ml of phenolics to various seed oils would modify Nrf2 activation after a meal. They found no significant differences in Nrf2 gene expression 2 and 4 h post-intake but a ~ 18 to 25% decrease in Nrf2 content (nuclear fraction) 4 h post intake in the phenolic containing oils, compared to sunflower oil. Magbanua et al. [63] examined the effects of the carotenoid, lycopene (30 mg/day), and fish oil (3 g/day) on Nrf2 gene expression in prostate tissue from prostate cancer patients. After 12 weeks, they found a significant increase in Nrf2 activation after both supplements compared to a placebo control. Yubero-Serrano et al. [64] reported a significant increase in cytosolic Nrf2 content (4 h post-food intake) in healthy individuals who consumed co-enzyme Q-10 (200 mg/day) alongside a Mediterranean diet for four weeks. In the same study, this intervention was also found to significantly reduce nuclear Nrf2 protein content and gene expression 4 h after a mixed macronutrient meal. A large randomized controlled trial [62] reported a significant increase in Nrf2 gene expression (~ 40%) and protein content (~ 30%) after a 24 week intake of isoflavones (80 mg/day) in ischemic stroke patients. Jackman et al. [52] found that a 2-week intake of anthocyanin rich cherry juice (60 ml/day) had no effect on Nrf2 gene expression in skeletal muscle from 16 healthy older adults. By contrast, four-week intake of an anthocyanin rich mixture of grape polyphenols increased Nrf2 gene expression (~ 1.2 fold) 3 h following a high fat, high carbohydrate meal [67].

### Risk of bias

**Fig. 4:**
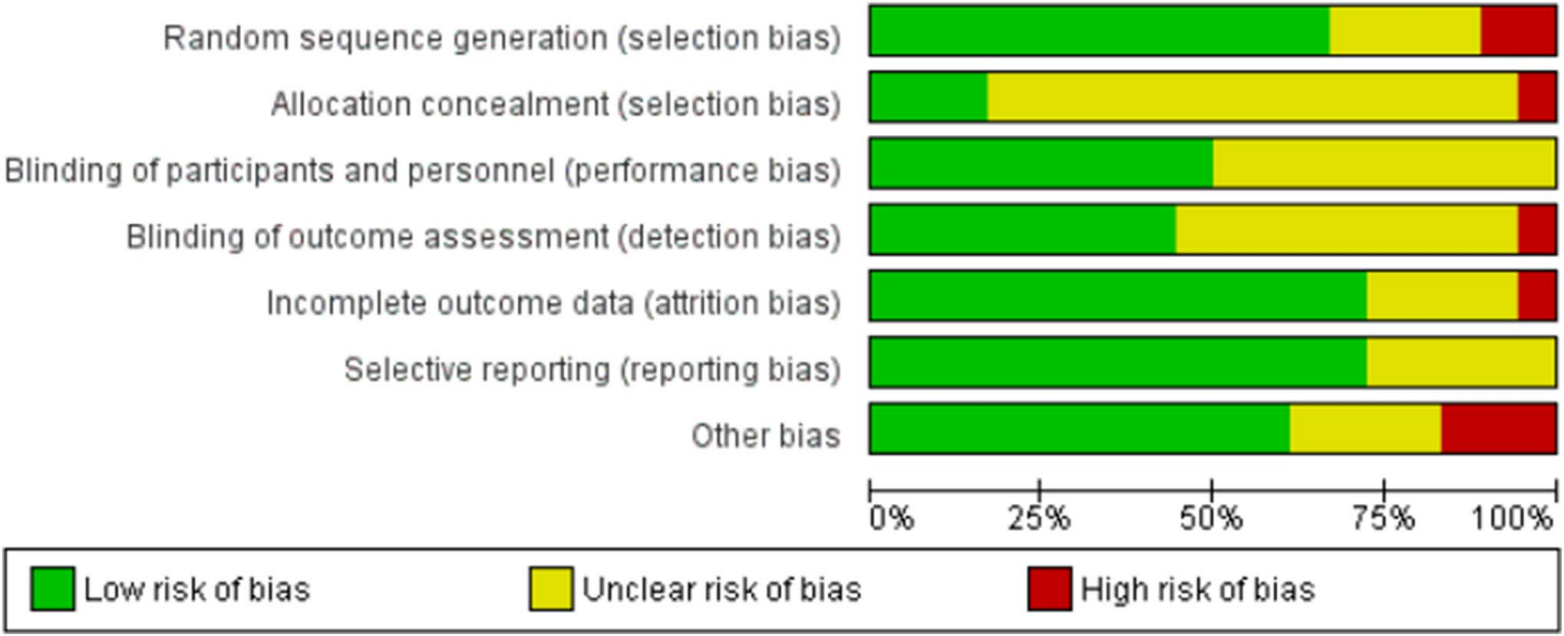
Risk of bias summary for individual studies

There was a large variation in study quality (Figs. 3 and 4). Only two [46, 58] of the eighteen trials were deemed to be sufficiently well conducted and reported to have a low risk of bias for all variables; neither of which reported an increase in Nrf2 activation after the intervention. Four studies had an unclear risk of bias for random sequence generation because of insufficient details were given or no randomization appeared to be performed [45, 54, 55, 66]. Two studies were deemed to have a high risk of bias for sequence allocation because they did not randomize the treatment order; all participants had the control first and then the intervention after a washout period [51, 61]. All but three studies [46, 57, 58] had an unclear or high risk of bias for allocation concealment because insufficient information was given. One study was deemed high risk for this variable because participants were told to avoid the intervention in the weeks leading up to the trial [55]. Nine studies had an unclear risk of bias for blinding of participants or personnel and blinding of outcome because insufficient details were provided [45, 51, 54, 55, 60–62, 65, 66]. One study had a high risk of bias because the assessors were not blinded to the intervention groups [59]. In four studies, it was unclear if there was attrition bias [51, 57, 61, 66]. One study was considered high risk because the number of participants enrolled did not match the number reported for the outcomes and no explanation was provided for this discrepancy [60]. Five studies were judged to have an unclear risk of bias for selective reporting due to insufficient information [51, 55, 59, 61, 66]; two studies were deemed to have an unclear risk for other bias due to funding sources [45, 52] and two for a poorly described study design [51, 61]. Three studies were deemed to have a high risk for other bias because the studies had no comparator control [54, 55, 66].

## Discussion

This is the first study to systematically review trials examining the effects of phytochemicals on Nrf2 activation in humans. Across 18 studies, 12 different phytochemicals were examined, of which curcumin, resveratrol and sulforaphane were the most frequent. More than half the studies found evidence of a Nrf2 inducing effect (*n* = 10); however, many of them had a high risk of bias and were poorly controlled. Overall, this study found limited high-quality evidence of phytochemicals activating Nrf2 in humans.

There was wide heterogeneity in study quality in human trials assessing the influence of phytochemical administration on Nrf2 activation. Many studies had an unclear or high risk of bias as a result of inadequate randomization, allocation concealment and blinding procedures. Three trials had an unclear or high risk of bias for most variables as they did not include a comparator control arm and instead employed pretest posttest quasi experimental designs [54, 55, 66]. In all three studies, Nrf2 activation was measured as changes in gene expression before and after the intervention only. These changes also might not translate to increases in Nrf2 protein content. Another limitation in many studies was low sample size. Eight studies contained less than twenty participants, and only two conducted a power analysis for measuring Nrf2 [46, 58]. Many did not state a primary outcome and of those that did only two reported that it was Nrf2 [46, 58]. Thus, it would be reasonable to assume that many of the studies were not adequately powered to detect effects, and that the risk of type two errors was high. Another potential source of bias was the lack of dietary control in the studies. Habitual dietary intake is an important confounding factor when evaluating the nutrigenomic effects of phytochemicals [70]; however, it was largely ignored by the included studies. Many trials did not state whether participants continued with their normal diet or altered their intakes during the study duration, and only three studies provided a detailed analysis of participants dietary intake [52, 64, 67]. Finally, and as evident from Table 1, the reporting of methods was poor in some studies, with many providing inadequate information for replication. In summary, more appropriately powered studies are needed to evaluate the efficacy of dietary supplementation with phytochemicals to enhance Nrf2 activation with Nrf2 as the primary outcome and, where possible, such studies should be conducted according to the Consolidated Standards of Reporting Trials (CONSORT) guidelines [71] so the highest quality evidence is obtained and reported.

In numerous in vitro studies, curcumin [72, 73] and resveratrol [74–76] have been shown to induce Nrf2 activation. Therefore, it is perhaps unsurprising that these were found to be the most studied polyphenols in humans. Curcumin has a Michael acceptor in the form of a α,β-unsaturated carbonyl group and thus the main mechanism by which it activates Nrf2 is by alkylating a protein thiol on the Keap-1-Nrf2 binding complex, which allows Nrf2 to translocate to the nucleus to initiate antioxidant gene expression changes [34, 38, 73, 77]. Resveratrol does not contain a Michael acceptor but instead likely acquires electrophilic and therefore Nrf2 inducing capacity through oxidation to a reactive quinone [38].

In the three studies that administered curcumin, two were shown to increase Nrf2 activation and one had no effect. However, as mentioned above, neither of the studies reporting positive effects were randomized, placebo-controlled trials and therefore had a high risk of bias [54, 55]. For example, in one of these studies [55], Nrf2 expression was measured in twelve healthy volunteers before and after 4 g of curcumin and a mixed macronutrient breakfast. While 4–6 h post Nrf2 gene expression was ~ 1.3 fold higher compared to baseline, there was no comparator group in this study and therefore it is not clear if this increase is a direct result of the curcumin. In contrast, the study by Jimenez and colleagues [56], was higher quality, employing a double blind, randomized, controlled design in a large cohort of diabetic patients. They found that eight weeks of curcumin (320 mg/day) had no effect on Nrf2 nuclear binding activity in PBMCs, despite curcumin lowering malondialdehyde, a marker of lipid peroxidation. This latter finding suggests that Nrf2 activation was not responsible for the lowering of oxidative stress and there must be other mechanisms by which curcumin functions as an antioxidant, perhaps by inhibiting oxidant producing immune cells [78, 79]. Nevertheless, a decrease in only one marker (malondialdehyde) is not deemed sufficient to verify oxidative stress [80]; therefore, these results should not be interpreted as evidence of a strong antioxidant effect [20, 81]. In summary, only one randomized controlled trial has examined the influence of curcumin administration on Nrf2 activation in humans and the results suggest that it was ineffective. Thus, there is currently no high-quality evidence showing that curcumin administration actives Nrf2 in humans.

Three studies examined the effects of resveratrol on Nrf2 activation. Two of these reported that resveratrol increased Nrf2 activation, either via DNA binding activity after a single dose [45] or via gene expression after two months of intake [57]. It should be noted that, in the former study, resveratrol was administered alongside 75 mg of undisclosed grape polyphenols and thus these effects cannot necessarily be ascribed to resveratrol. Confusingly, the findings in the latter study [57] do not match how the data was interpreted; the authors state that resveratrol increased Nrf2 expression and discuss these findings accordingly, yet the data they present shows that resveratrol decreased Nrf2. The authors were contacted to clarify these findings, but no response was received. In contrast to these studies, a study with 20 chronic kidney disease patients found that four weeks of resveratrol (500 mg/day) had no effect on Nrf2 gene expression in PBMCs [58]. To explain their null findings, the authors speculated that the dose was too low, citing that the bioavailability of resveratrol was probably compromised in patients with renal impairments. However, they did not measure tissue levels of resveratrol or its metabolites to confirm this supposition. Overall, there is weak evidence that resveratrol increases Nrf2 activation in humans and therefore more high-quality research is required to corroborate or refute the in vitro and animal data.

In three studies the principal compound administered was sulforaphane, an isothiocyanate phytochemical present in cruciferous vegetables such as broccoli [82]. From a mechanistic perspective, sulforaphane is an electrophile that can synthesize Nrf2 by reacting with the sulphur-rich cysteine residues on its repressor Keap-1 [83, 84]. Sulforaphane is perhaps the most well studied Nrf2-activating compound to date, partly owing to its superior bioavailability to other phytochemicals [82, 85]. There are now several clinical trials showing that sulforaphane attenuates inflammation and upregulates Nrf2 target genes in a range of pathologies [86–88]. Intriguingly, these effects are largely ascribed to Nrf2 activation, yet we found little evidence that sulforaphane activates Nrf2 in humans. In the one study reporting positive effects [59], the findings were biased by a low sample size (4 subjects) and inadequate blinding of assessors. In addition, the authors did not state whether the increase in Nrf2 expression was statistically significant. In contrast, a broccoli sprout homogenate that contained an undisclosed amount of sulforaphane, and possibly other bioactive compounds, had no effect on gene expression in PBMCs or nasal epithelial cells (Duran et al. 2016). This study was also hampered by a small sample size (*n* = 15). Nonetheless, their findings are consistent with a well-controlled clinical trial that found no effects of sulforaphane on Nrf2 activation in chronic obstructive pulmonary disease patients [46]. In this study, the authors speculated that persistent redox and immune dysregulation in this population could have rendered the intervention less effective. This postulate is inconsistent with studies that found sulforaphane upregulated Nrf2 target genes in type 2 diabetics [88] and obese patients [87], who also have high levels of oxidative stress and low grade inflammation. However, as these studies did not measure Nrf2, it is possible that the cytoprotective effects reported with sulforaphane were independent of the Nrf2-ARE pathway. There is evidence that sulforaphane inhibits inflammation by blunting the pro-inflammatory transcription factor nuclear factor kappa B (NF-κB), which provides some support for Nrf2-ARE independent effects of sulforaphane administration [89]. In summary, we found little evidence that sulforaphane activates Nrf2 in humans. Since there are several ongoing clinical trials with sulforaphane containing supplements, evidence to support the efficacy of sulforaphane as a Nrf2 activator in humans will hopefully become clearer over the next few years.

Two studies reported that daily intake of 500–750 ml of coffee made from beans enriched with chlorogenic acid or n-methylpyridinium increased Nrf2 gene expression in healthy young adults [51, 61]. Despite these positive findings, it is important to note that both studies had a high risk of bias on the bases that no randomization was performed, there was no mention of double blinding, and the comparator arm was a low polyphenol diet with no information provided on what constituted a low polyphenol diet. Thus, while these studies are promising, well-controlled randomized trials are needed to confirm the efficacy of coffee consumption to increase Nrf2 activation.

The duration of supplementation varied widely across all studies, and there did not seem to be any specific pattern to discern the optimal length of the intervention. Indeed, Nrf2 activation increased with a single dose [45] or after several weeks of intake in others [62, 63]. There is presently no consensus as to what the optimal dose or duration is for activating Nrf2 with phytochemicals or drugs [30, 82]. The optimal dose will not only depend on the quantity administered, but on its bioavailability, concentration reached in target cells, and the patients’ age and health status [30]. Well-controlled, multiple dose, pharmaco-kinetic studies in a variety of patient groups and tissues will be needed to acquire this knowledge.

It has been shown that Nrf2 activation declines with age [90, 91] and is downregulated in diseases such as type 2 diabetes [92] and atherosclerosis [93]. In view of this, it could be speculated that older, diseased individuals are more likely to benefit from an intervention attempting to reestablish Nrf2 activation than young, healthy individuals, in whom Nrf2 activation is unlikely to be impaired. However, this might not be the case for patients on some medications. Indeed, it would be reasonable to assume that some medications which modulate redox signaling might interfere with Nrf2 activation, in which case it would be difficult to determine the independent effect of a phytochemical intervention on this pathway in some patient populations. Notwithstanding these potential confounders, this review found no clear evidence that age, health or medications modified the efficacy of the phytochemical interventions on Nrf2 activation. Indeed, as shown in Table 1, findings in young and healthy, or diseased individuals, were equally mixed, such that no discernable pattern emerged to suggest better or worse efficacy in a specific population or in those on concomitant medications. While Nrf2 was activated with isoflavones in stroke patients (almost half of whom were on medication) [62] and fish oil and lycopene in prostate cancer patients [63], in what were large relatively well controlled trials, it would be premature to suggest these populations could benefit from these interventions based on findings from isolated studies. Equally, the null effects of curcumin in diabetic patients [56] or sulforaphane in chronic obstructive pulmonary disease patients [46] should be not seen as definitive evidence that they are ineffective in these diseases. Ultimately, there were too few studies in any one population to discern what diseases or conditions these phytochemicals can or cannot modify Nrf2 activation.

Interestingly, in three studies there was evidence that phytochemicals decreased Nrf2 activation [64–66]. These studies differed markedly in terms of study design, participants and compounds used (bilberry pomace, co-enzyme Q10, phenolic-rich seed oils) and thus an explanation for their effects is unclear. Many phytochemicals have anti-inflammatory effects [94] and therefore one possible explanation is that these compounds attenuated immune cell derived reactive species that activate Nrf2. However, this was not explored in either study. It is also possible that these phytochemicals promoted Nrf2 degradation; somewhat paradoxically, there is in vitro evidence that certain flavonoids can inhibit Nrf2 activation [95]. However, these observations remain contentious and the mechanisms have not been elucidated [96]. The ability of different phytochemicals to attenuate Nrf2 activation clearly requires further research.

## Recommendations for future research

Based on the findings of this review, a number of recommendations for future studies can be provided. Ideally, future trials will be randomized, placebo controlled and have a low risk of bias, like some in this review [46, 58, 63]. Although most phytochemicals are generally recognized as safe, it is important that trials record any adverse effects so that dosing regimens can be modified if necessary. These studies are required before titration studies to determine optimal doses and durations are considered. Trials are needed in both healthy and diseased populations of different ages, and confounding factors such as dietary intake, medication, ethnicity, body mass, and physical activity levels should be accounted for in randomization and analysis. Stage of disease progression is also another important factor to consider. This is highlighted by the ongoing work in cancer, which suggests that Nrf2 can have both a positive and detrimental role in cancer survival depending on the stage of the disease [96, 97]. In terms of measurement, Nrf2 activation can be evaluated in PBMCs and other accessible tissues (nasal cells etc.) with relative ease through gene expression and/or protein content assays. An obvious caveat is that these cells might not reflect changes in the target tissue. However, at least in PBMCs, changes in immune and redox signaling have been shown to strongly correlate with changes in several organs [98, 99]. Furthermore, Nrf2 is highly expressed in blood cells and therefore easily detectable [30]. A discussion of the methods used to quantify Nrf2 in these matrices is beyond the scope of this review but measuring total and phosphorylated Nrf2 in nuclear and cytosolic fractions at the gene and protein level would provide the most comprehensive data. It might also be useful to evaluate Nrf2 effects at multiple time-points to avoid missing any transient changes. In the present review, single dose studies showed that changes in gene and protein expression were not evident until 3–5 h post-intake and thus the timing of Nrf2 measurement might be an important consideration for future research. It would also be useful to measure Nrf2 activation alongside downstream genes such as superoxide dismutase, catalase, glutathione peroxidase, glutathione *s*-transferase, heme oxygenase 1, glutathione and thioredoxin, which participate in cellular defense and have longer half-lives than Nrf2 [30]. Various phytochemicals have been shown to upregulate these and other Nrf2 target genes in humans [86–88, 100, 101]; however, as highlighted by this review, few studies also include measures of Nrf2, meaning these effects cannot be causally linked to activation of the Nrf2-ARE pathway. Many of the target genes such as heme oxygenase 1 and catalase can be upregulated by Nrf2-independent pathways [102–105], which could explain, at least in part, why some of the included studies report a disparity in the activation of Nrf2 and Nrf2 target genes [51, 57, 64–66]. In addition, systemic concentrations of the phytochemicals administered and where relevant its metabolites should be measured using appropriate techniques (e.g., liquid chromatography–mass spectroscopy). The bioavailability of the dietary phytochemicals was overlooked by almost all studies in this review and should be prioritized in future trials. Finally, future studies should explore the potential synergistic, additive or antagonistic effects of different phytochemical combinations on Nrf2 activation. There is growing evidence that the biological activity of some phytochemicals, such as curcumin, is augmented by the addition of piperine, partly as a result of improved bioavailability [106, 107]. Combinations such as these are worth exploring in future studies. Collectively, the outlined measures will help to ensure the data collected is high quality and able to advance our current understanding of how dietary phytochemical interventions modulate Nrf2 activation in humans.

## Conclusions

The current review provides a systematic summary of the evidence for administration of dietary phytochemicals to induce Nrf2 in humans. According to our review, there is insufficient high-quality evidence indicating that phytochemicals activate Nrf2 in humans. While many phytochemicals increased Nrf2 activation in single studies, it would be premature to single out any one specific compound due to the overall paucity of well controlled clinical trials and the diverse findings reported. Of critical importance moving forward is that future studies examine if the positive findings reported in cell culture and animal studies are translatable to humans. The pleiotropic role of Nrf2 in modulating system wide cytoprotective defenses means targeting this single transcription factor has the potential to improve health outcomes in a myriad of pathologies. While phytochemicals are unlikely to be as potent as the pharmacological agents currently being developed to activate Nrf2, they could play an important role as cost-effective, complementary, or preventative therapies. We hope these findings provide researchers with the impetus to conduct high quality human intervention studies examining the effects of dietary phytochemicals on Nrf2 activation.

## Supplementary information

Below is the link to the electronic supplementary material.

(DOCX 22 kb)
