# Supplementary material for: The effect of dietary phytochemicals on nuclear factor erythroid 2-related factor 2 (Nrf2) activation: a systematic review of human intervention trials
Source: Mol Biol Rep. 2021 Jan 30;48(2):1745–61. doi: 10.1007/s11033-020-06041-x (PMC7925463; doi:10.1007/s11033-020-06041-x)
Supplement: Supplementary file 1 — (DOCX 22 kb) [file 11033_2020_6041_MOESM1_ESM.docx]

**Online Supplementary Material**

Search strategy: MEDLINE, EMBASE, CAB Abstracts

01. phytochemical*

02. dietary phytochemical*

03. plant bioactive*

04. nutraceutical*

05. plant derived compound*

06. plant derived chemical*

07. polyphenol*

08. phenol*

09. (poly)phenol

10. phytonutrient*

11. lignan*

12. flavan-3-ol

13. terpene*

14. carotenoid*

15. limonoid *

16. phytosterol*

17. thiol*

18. glucosinolate*

19. indole*

20. isoprenoid*

21. lipoic acid*

22. ubiquinone

23. flavonoid*

24. flavanol*

25. tannin*

26. stilbene*

27. isoflavone*

28. anthocyani*

29. proanthocyani*

30. flavanone*

31. flavonol*

32. flavone*

33. allicin

34. capsaicin

35. catechin*

36. ellagic acid

37. genistein*

38. lycopene*

39. saponin*

40. zeaxanthin*

41. curcumin*

42. resveratrol*

43. naringin*

44 epigallocatechin-3-gallate*

45. caffeic acid*

46.sulforaphane*

47. 1 or 2 or 3 or 4 or 5 or 6 or 7 or 8 or 9 or 10 or 11 or 12 or 13 or 14 or 15 or 16 or 17 or 18 or 19 or 20 or 21 or 22 or 23 or 24 or 25 or 26 or 27 or 28 or 29 or 30 or 31 or 32 or 33 or 34 or 35 or 36 or 37 or 38 or 39 or 40 or 41 or 42 or 43 or 44 or 45 or 46

48. nuclear factor erythroid-derived 2-like 2*

49. nuclear factor erythroid-derived 2*

50. nuclear factor erythroid derived 2*

51. nuclear factor erythroid 2 related factor 2*

52. nuclear factor erythroid 2-related factor 2*

53. nf-e2-related factor 2

54. nuclear erythroid 2 p45-related factor*

55. nuclear erythroid 2 p45 related factor*

56. nrf2

57. nrf2-ARE

58. nfe2l2

59. nfe2l

60. nf-e2-related factor 2

61. 48 or 49 or 50 or 51 or 52 or 53 or 54 or 55 or 56 or 57 or 58 or 59

62. 47 and 61

63. exp animals/ not humans

64. 62 not 63

65. 64 and 62

| Table S1: PICOS methodology | |
| --- | --- |
| Population | Adult human participants (≥18 years); no exclusion criteria were applied in relation to participants’ health status. |
| Intervention | Supplementation with a phytochemical: studies were included if they provided information on the type of compound(s), and the dose, frequency and route of administration. |
| Comparator | A control or placebo group or to baseline values in quasi-experimental design. |
| Outcome | Trials reporting the effects of a phytochemical (or a combination of) on Nrf2. No exclusion criteria were employed for the methods used to quantify Nrf2. |
| Study design | No exclusion criteria were used for study design; human trials only, animal data was excluded. |

**References for chemical structures in Figure 2**

| 2D structure image of CID 445154 (Resveratrol) | https://pubchem.ncbi.nlm.nih.gov/compound/445154 |
| --- | --- |
| 2D structure image of CID 969516 (Curcumin) | https://pubchem.ncbi.nlm.nih.gov/compound/969516 |
| 2D structure image of CID 5350 (Sulforaphane) | https://pubchem.ncbi.nlm.nih.gov/compound/5350 |
| 2D structure image of CID 70267806 (Soybean Isoflavones) | https://pubchem.ncbi.nlm.nih.gov/compound/70267806 |
| 2D structure image of CID 5281915 (Coenzyme Q10) | https://pubchem.ncbi.nlm.nih.gov/compound/5281915 |
| 2D structure image of CID 5281915 (Lycopene) | https://pubchem.ncbi.nlm.nih.gov/compound/446925 |
| 2D structure image of CID 446284 (Eicosapentaenoic acid) | https://pubchem.ncbi.nlm.nih.gov/compound/446284 |
| 2D structure image of CID 445580 (Docosahexaenoic acid) | https://pubchem.ncbi.nlm.nih.gov/compound/445580 |

**List of studies for which full texts were retrieved but were excluded**

1. Appendino G, Belcaro G, Cornelli U, et al. Potential role of curcumin phytosome (Meriva) in controlling the evolution of diabetic microangiopathy. A pilot study. *Panminerva Med*. 2011.

2. Brown RH, Reynolds C, Brooker A, Talalay P, Fahey JW. Sulforaphane improves the bronchoprotective response in asthmatics through Nrf2-mediated gene pathways. *Respir Res*. 2015. doi:10.1186/s12931-015-0253-z

3. Carrizzo A, Puca A, Damato A, et al. Resveratrol improves vascular function in patients with hypertension and dyslipidemia by modulating NO metabolism. *Hypertension*. 2013. doi:10.1161/HYPERTENSIONAHA.111.01009

4. Crespo MC, Tomé-Carneiro J, Burgos-Ramos E, et al. One-week administration of hydroxytyrosol to humans does not activate Phase II enzymes. *Pharmacol Res*. 2015. doi:10.1016/j.phrs.2015.03.018

5. González-Guardia L, Yubero-Serrano EM, Delgado-Lista J, et al. Effects of the mediterranean diet supplemented with coenzyme Q10 on metabolomic profiles in elderly men and women. *Journals Gerontol - Ser A Biol Sci Med Sci*. 2015. doi:10.1093/gerona/glu098

6. Heber D, Li Z, Garcia-Lloret M, et al. Sulforaphane-rich broccoli sprout extract attenuates nasal allergic response to diesel exhaust particles. *Food Funct*. 2014. doi:10.1039/c3fo60277j

7. Noah TL, Zhang H, Zhou H, et al. Effect of broccoli sprouts on nasal response to live attenuated influenza virus in smokers: A randomized, double-blind study. *PLoS One*. 2014. doi:10.1371/journal.pone.0098671

8. Pastorelli D, Fabricio ASC, Giovanis P, et al. Phytosome complex of curcumin as complementary therapy of advanced pancreatic cancer improves safety and efficacy of gemcitabine: Results of a prospective phase II trial. *Pharmacol Res*. 2018. doi:10.1016/j.phrs.2018.03.013

9. Turowski JB, Pietrofesa RA, Lawson JA, Christofidou-Solomidou M, Hadjiliadis D. Flaxseed modulates inflammatory and oxidative stress biomarkers in cystic fibrosis: A pilot study. *BMC Complement Altern Med*. 2015. doi:10.1186/s12906-015-0651-2

10. Yanaka A. Sulforaphane Enhances Protection and Repair of Gastric Mucosa Against Oxidative stress In Vitro, and Demonstrates Anti-inflammatory Effects on Helicobacter pyloriInfected Gastric Mucosae in Mice and Human Subjects. *Curr Pharm Des*. 2011. doi:10.2174/138161211796196945

11. Yanaka A, Fahey JW, Fukumoto A, et al. Dietary sulforaphane-rich broccoli sprouts reduce colonization and attenuate gastritis in Helicobacter pylori-infected mice and humans. *Cancer Prev Res*. 2009. doi:10.1158/1940-6207.CAPR-08-0192
